# Growth Differentiation Factor 15 Predicts Cancer Death in Patients With Cardiovascular Risk Factors: The J-HOP Study

**DOI:** 10.3389/fcvm.2021.660317

**Published:** 2021-06-04

**Authors:** Keita Negishi, Satoshi Hoshide, Masahisa Shimpo, Kazuomi Kario

**Affiliations:** ^1^Division of Cardiovascular Medicine, Department of Medicine, Jichi Medical University, Tochigi, Japan; ^2^Jichi Medical University Center of Excellence, Community Medicine Cardiovascular Research and Development, Tochigi, Japan

**Keywords:** GDF-15, cancer, mortality, cardiovascular disease, onco-cardiology

## Abstract

**Background:** Disease-related anorexia-cachexia is associated with poor prognosis of patients with cardiovascular disease (CVD) or cancer. Growth differentiation factor-15 (GDF-15) has emerged as a central regulator of appetite and body weight. However, the exact role of GDF-15 in lean patients has not been elucidated.

**Aim:** Our aim is to evaluate whether the association of GDF-15 with mortality, including cancer death, differs according to body mass index (BMI) level.

**Methods and Results:** We collected blood samples from 4,061 patients with CV risk factors who were enrolled in the nationwide practice-based J-HOP (Japan Morning Surge-Home Blood Pressure) study. Serum GDF-15 levels were determined by immunoassay analysis. During a mean follow-up period of 6.6 years, we observed 174 (6.7/1000 person-year) all-cause deaths, 68 (2.6/1000 person-year) cancer deaths, and 56 (2.2/1000 person-year) CV deaths. Patients were stratified according to the cut-points of GDF-15 at 1,200 ng/L and BMI at 22.5 and 25.0 kg/m^2^. The association between the GDF-15/BMI based study groups and each outcome was evaluated by Cox-proportional hazard models with adjustment for established risk factors. The multivariate Cox regression model showed that patients with elevated GDF-15 (≥1,200 ng/L) and low BMI (<22.5 kg/m^2^) were significantly associated with increased risk of all outcomes [all-cause death, hazard ratio (HR) 3.15, 95% confidence interval (CI) 1.85–5.34, *p* < 0.001; cancer death, HR 3.52, 95%CI 1.64–7.57, *p* = 0.001; CV death, HR 2.88, 95%CI 1.20–6.92, *p* = 0.018, respectively] compared to a reference group with non-elevated GDF-15 and normal BMI (22.5–25.0 kg/m^2^). In analyses of a subgroup with low BMI (<22.5 kg/m^2^), patients with elevated GDF-15 had 4.79-fold increased risk of cancer death and 11-fold greater risk of CV death when compared with patients with non-elevated GDF-15 (<1,200 ng/L) after adjustment for established risk factors.

**Conclusion:** In patients with CV risk factors, GDF-15 was associated with all-cause, cancer, and CV death. This relationship was especially remarkable in patients with low BMI. The serum GDF-15 levels in patients with low BMI might be a useful marker to identify the potential for anorexia-cachexia associated with CVD and cancer.

## Introduction

Cardiovascular disease (CVD) is the main cause of mortality globally, but cancer is a more common cause of death than CVD in some high-income and upper-middle-income countries (1). In these countries, as longevity has increased due to advances in medical care, the number of patients having both CVD and cancer has also increased, and onco-cardiology has emerged as a new discipline for the clinical management of these patients. Recent efforts have focused on disease prevention as well as treatment of cancer therapeutics-related cardiac dysfunction. In addition, there are a number of shared risk factors and common pathologies regulating the prognosis of CVD and cancer (2).

Growth differentiation factor-15 (GDF-15), a member of the transforming growth factor-beta superfamily (3), is expressed in multiple organs in response to tissue stress in many disease states (4, 5). Higher levels of GDF-15 may be useful for the diagnosis and management of CVD (4, 5) and cancer (6, 7) and are a strong predictor of mortality from either disease (8–10). On the other hand, it has been reported that GDF-15 exerts a cardioprotective effect through the activation of anaplastic lymphoma kinase (ALK) receptors and phosphorylation of the Smad signaling pathway (11) and that it has an overall protective role in early cancer (6). Serum GDF-15 levels might be elevated in patients with CVD and cancer compensatorily. Furthermore, emerging evidence indicates that GDF-15 regulates body weight through an effect on the appestat (12–15). GDF-15 forms a co-receptor complex with glial cell-derived neurotrophic factor receptor alpha-like (GFRAL) and rearranged during transfection (RET) and induces an anorexia effect *via* the appestat (12–15) independently of other well-known appetite-related pathways involving melanocortin-4, leptin, or incretin (13). Weight loss in patients with CVD or cancer—i.e., disease-related anorexia-cachexia—is a well-known phenomenon and clearly associated with poor prognosis. In previous cohorts, patients with chronic kidney disease (16), heart failure (17), and cancer (18) also showed an inverse correlation between serum GDF-15 levels and body mass index (BMI). However, in general outpatients under potential stress, it is not clear whether GDF-15 is a driver of the association between lower BMI and adverse outcome. In this study, we evaluated the prognostic value of GDF-15 and the relationship between GDF-15 and BMI in a large general practice population of patients with CV risk factors.

## Methods

### Study Design

All subjects were recruited from the Japan Morning Surge-Home Blood Pressure (J-HOP) study (19). The J-HOP study was a nationwide prospective study conducted in Japan that included 4,310 outpatients with any of the following risk factors for CVD: hypertension (clinic systolic blood pressure [SBP] >140 mmHg and/or diastolic blood pressure [DBP] >90 mmHg, or current use of antihypertensive medication), hyperlipidemia (total cholesterol level >240 mg/dl or treated hyperlipidemia), diabetes mellitus (fasting blood sugar ≥126 mg/dL or current use of an antidiabetic drug), glucose intolerance (fasting blood sugar >140 mg/dl at 2 h after a 75-g oral glucose tolerance test), metabolic syndrome (20), chronic kidney disease (estimated glomerular filtration rate [eGFR] <60 mL/min/1.73 m^2^), history of CVD (CAD, stroke, aortic dissection, peripheral artery disease, or congestive heart failure), atrial fibrillation, current smoking, chronic obstructive pulmonary disease, and sleep apnea syndrome (an apnea hypopnea index of >15 events/hour by overnight sleep polysomnography).

The exclusion criteria for the J-HOP Study were a recent history of cardiovascular disease events (within the most recent 6 months), malignancy, current hemodialysis treatment, and chronic inflammatory disease. Patients were recruited for the J-HOP study between 2005 and 2012 and followed up through March 2015 by 75 doctors at 71 institutions (45 primary practices, 22 hospital-based outpatient clinics, and 4 specialized University hospitals). The study protocol was registered on the University Hospital Medical Information Network Clinical Trials Registry (UMIN000000894). All participants provided written informed consent, and the Institutional Review Board of Jichi Medical School approved the study.

### Laboratory Testing

The J-HOP study personnel collected blood samples from the patients at enrollment after they had completed a fasting period. The blood samples were centrifuged at 3,000 g for 15 min at room temperature. The supernatants were stored at 4°C, sent to a commercial laboratory (SRL Inc., Tokyo), frozen in aliquots, and stored at −80°C in a deep freezer. All routine biochemical analyses were performed within 24 h of sample collection at this single laboratory center.

The patients' GDF-15 levels were measured with an automated platform (Cobas e 411 analyzer; Roche Diagnostics, Indianapolis, IN). The assay has a limit of detection below 400 ng/L, a linear measuring range up to 20,000 ng/L, and an inter-assay imprecision of 2.3% and 1.8% at GDF-15 concentrations of 1,100 and 17,200 ng/L, respectively (Roche Diagnostics; data on file).

### Study Groups Based on GDF-15 and BMI

Study participants were categorized into six groups based on a combination of GDF-15 and BMI. For GDF-15, a serum level ≥1,200 ng/L was defined as elevated (21, 22). In accordance with the large-scale Asian cohorts (23, 24), a range of BMI from 22.5 to 25.0 kg/m^2^ was defined as normal because this range is associated with the lowest risk of mortality. The six GDF-15/BMI-based study groups were thus as follows: Group A, non-elevated GDF-15 (<1200 ng/L) with normal BMI (22.5–25.0 kg/m^2^); Group B, non-elevated GDF-15 with high BMI (≥25.0 kg/m^2^); Group C, non-elevated GDF-15 with low BMI (<22.5 kg/m^2^); Group D, elevated GDF-15 (≥1200 ng/L) with normal BMI; Group E, elevated GDF-15 with high BMI; and Group F, elevated GDF-15 with low BMI.

### Outcome Ascertainment

The outcomes were categorized as follows: (1) all-cause death; (2) cancer death defined as *de novo* cancer-related mortality; (3) CV death defined as death from cardiovascular disease (i.e., acute myocardial infarction, cerebrovascular attack, heart failure) and including sudden death. The criteria for myocardial infarction included definite electrocardiographic findings (i.e., ST elevation), typical or atypical symptoms together with electrocardiographic findings and abnormal enzymes, or typical symptoms and abnormal cardiac enzymes with or without electrocardiographic findings. Cerebrovascular attack included cerebral infarction, cerebral hemorrhage, and subarachnoid hemorrhage based on the findings of brain CT or MRI. Transient ischemic attack was not included.

Evidence on the above death outcomes was ascertained by ongoing reports from a general physician at each institute. The cause of death was also ascertained by means of frequent reviews of patients' medical records. When patients failed to come to the hospital, their family members were interviewed by telephone. The end point committee adjudicated all events by reviewing the patients' files and source documents and by requesting more detailed written information from investigators when necessary.

### Statistical Analyses

Demographics and other baseline characteristics were compared across the GDF-15/BMI-based study groups. Continuous variables are presented as medians and interquartile ranges (IQRs), and groups were compared using the Kruskal-Wallis test. Categorical variables are presented as counts and percentages, and groups were compared using χ^2^ tests. GDF-15 values were logarithmically transformed due to skewed distributions. Blood concentrations under the measuring limit of each biomarker were calculated as the half-value of the limit, i.e., GDF-15 at 200 ng/L.

Unadjusted risks of outcomes were assessed across the BMI/biomarker-based study groups using cumulative incidence plots and log-rank tests. We evaluated the association between baseline GDF-15 levels and outcomes by using multivariable Cox proportional hazards models with a log-transformed continuous model and with a dichotomous model (relative to under 1,200 ng/L at GDF-15). Multivariable adjusted analysis was also performed to assess the association between the GDF-15/BMI-based study groups and risk of outcomes. By way of sensitivity analyses, we analyzed the relation of GDF-15 level to outcome in three subgroups based on the cut-point of BMI (22.5 and 25.0 kg/m^2^). Hazard ratios (HRs) were calculated after a composite risk score, renal function (estimated glomerular filtration rate [eGFR]), daily drinker (as a factor affecting body weight), and inflammation (high sensitive C-reactive protein [hs-CRP]). Composite risk scores are a useful approach for controlling for confounders because of concerns of overfitting the models as a result of the limited number of events among the study participants. The composite risk scores were created in the study population by determining the 10-year predicted probabilities for each outcome using Cox proportional hazards regression models, including the covariates of CV traditional risk factors (i.e. age, sex, current smoking, diabetes mellitus [DM], previous CV event, statin use, anti-hypertensive drug use, total cholesterol [TC], high-density lipoprotein cholesterol [HDL-C], office systolic blood pressure [SBP]) (25). We also analyzed with the individual covariates as sensitive analyses and showed the results in Supplementary Material. HRs and 95% confidence intervals (CIs) were expressed per 1 SD increase in GDF-15 level or relative to the reference group, respectively.

We analyzed the additional contribution of GDF-15 beyond traditional risk factors in predicting each outcome by using multiple metrics of biomarker performance, including discrimination (Harrell's c-statistics) (26) and reclassification [net reclassification index [NRI] (27) and integrated discrimination index [IDI] (28)]. Because no established categories exist that guide clinical decisions in relation to risk of cancer death with cardiovascular risk factors, we calculated a category-free NRI from time-dependent models. For the reclassification analyses, we estimated risk at 10 years. The 95%CI of each metric was estimated by using 1,000 bootstrap samples. Two-sided *p*-values < 0.05 were considered significant. All analyses were performed by using R, version 3.6.0 (The R Foundation for Statistical Computing) and SAS, version 9.4 (SAS Institute Inc).

## Results

### Baseline Characteristics

Among the 4,310 patients who were enrolled in the J-HOP study, the following were excluded: 221 patients whose blood samples were not sufficient for the measurement of the GDF-15 level, and 27 patients whose data were incomplete. The data of the final total of 4,061 patients were included in the analyses.

Table 1 provides the baseline clinical characteristics of the overall population and the patients as divided by the GDF-15/BMI-based study groups. In the overall population, the median age of the patients was 66 years, and there were more females than males. The median concentration of GDF-15 was 957.7 ng/L (IQR 700.0–1337.0 ng/L). The proportion of females was high at 56.6% in patients with GDF-15 <1200 ng/L, whereas the proportion of males was high (53.3%) in those with elevated GDF-15 (*p* < 0.001 by chi-square test). Age, office SBP, proportion of prior CVD, and smoking rates were higher, and eGFR tended to be low in patients with elevated GDF-15. Most of the patients had hypertension and were taking anti-hypertensive drugs, and the proportions of both hypertension and anti-hypertensive drug use were higher in patients with higher BMI. Higher TG and lower HDL-C concentration were also observed in patients with higher BMI.

**Table 1:** Baseline characteristics.

**Variable**	**Overall**	**Non-elevated GDF-15 with normal BMI**	**Non-elevated GDF-15 with high BMI**	**Non-elevated GDF-15 with low BMI**	**Elevated GDF-15 with normal BMI**	**Elevated GDF-15 with high BMI**	**Elevated GDF-15 with low BMI**	***p*-value**
		**(Group A)**	**(Group B)**	**(Group C)**	**(Group D)**	**(Group E)**	**(Group F)**	
*n*	4061	885	1035	834	428	459	420	–
GDF-15, ng/L	957.6	781.3	773.0	812.7	1582.0	1592.0	1625.0	–
	(699.7–1337.0)	(604.4–963.3)	(602.0–955.1)	(639.6–989.5)	(1344.0–2021.0)	(1353.0–2081.0)	(1373.0–2064.0)	
BMI, kg/m^2^	24.0 (22.0–26.1)	23.8 (23.2–24.4)	26.9 (25.9–28.7)	21.1 (19.9–21.9)	23.8 (23.2–24.4)	27.1 (25.9–29.0)	20.9 (19.8–21.8)	–
Age, y	65 (58–73)	62 (56–68)	60 (53–67)	64 (57–71)	73 (66–79)	71 (64–76)	74 (69–79)	<0.001
Male, %	46.6	47.7	46.7	35.0	55.6	48.2	56.7	<0.001
Prior CVD, %	13.8	11.2	10.9	10.1	20.6	23.1	16.9	<0.001
Office SBP, mmHg	140.0	139.3	139.0	139.0	143.0	141.8	141.5	<0.001
	(130.0–151.0)	(130.0–149.5)	(129.7–149.7)	(129.3–149.8)	(131.7–154.9)	(131.5–152.3)	(129.3–153.5)	
Office DBP, mmHg	80.8	82.7	83.7	80.4	78.3	79.0	76.8	<0.001
	(74.3–87.7)	(75.7–89.0)	(77.2–90.3)	(74.3–87.2)	(70.8–84.8)	(73.1–85.3)	(69.6–84.0)	
Current smoking, %	12.2	13.8	10.9	9.2	13.6	12.0	16.4	0.003
Daily drinker, %	27.3	29.5	27.3	26.5	28.5	23.5	27.1	0.304
Hypertension, %	91.3	91.1	93.4	85.6	93.7	95.9	90.7	<0.001
Diabetes mellitus, %	24.5	19.2	25.8	18.4	27.1	38.1	26.9	<0.001
Dyslipidemia, %	41.1	41.6	46.2	39.6	37.4	45.1	29.8	<0.001
Anti-hypertensive drugs, %	79.1	77.4	80.0	69.9	86.0	90.2	79.1	<0.001
Statin, %	23.6	21.9	26.5	23.3	20.6	29.0	18.1	<0.001
eGFR, mL/min/1.73m^2^	73.4	77.4	77.0	76.8	63.6	62.6	67.3	<0.001
	(62.5–84.5)	(66.8–86.7)	(67.8–88.0)	(67.4–86.6)	(51.5–76.1)	(49.9–73.7)	(56.0–76.8)	
Triglyceride, mg/dl	104 (76–149)	106 (78–155)	122 (87–172)	87 (67–116)	109 (79–157)	117 (88–167)	86 (66–120)	<0.001
Total cholesterol, mg/dl	201 (181–223)	205 (185–225)	204 (183–226)	206 (185–225)	195 (174–218)	193 (174–216)	192 (170–216)	<0.001
HDL-C, mg/dl	56 (47–66)	56 (48–66)	53 (45–62)	63 (54–75)	53 (44–62)	50 (42–60)	58 (47–69)	<0.001
HbA1c, %	5.7 (5.4–6.0)	5.6 (5.4–5.9)	5.7 (5.4–6.1)	5.6 (5.3–5.9)	5.7 (5.4–6.1)	5.9 (5.5–6.5)	5.6 (5.4–6.1)	<0.001
hs-CRP, mg/dl	522.0	993.3	674.0	332.5	564.0	844.0	467.5	<0.001
	(258.0–1130.0)	(253.0–916.0)	(350.5–1345.0)	(152.0–666.0)	(279.2–1232.5)	(389.0–1740.0)	(217.0–1130.0)	

*Continuous variables are presented as median (interquartile range, IQR), and categorized data are presented as number (percentage, %). GDF-15, growth differentiation factor-15; BMI, body mass index; CVD, cardiovascular disease; SBP, systolic blood pressure; DBP, diastolic blood pressure; eGFR, estimated glomerular filtration rate; HDL-C, high-density lipoprotein cholesterol; HbA1c, Hemoglobin A1c; hs-CRP, high sensitive C reactive protein. Prior CVD includes pre-existing angina pectoris, myocardial infarction, and stroke*.

### GDF-15 Predicted All-Cause, Cancer, and CV Death

During the mean follow-up of 6.6 ± 3.9 years, there were 174 all-cause deaths (6.7/1,000 person-years), of which 68 (2.6/1,000 person-years) were cancer deaths and 56 (2.2/1,000 person-years) were CV deaths. In patients with non-elevated GDF-15, the incidence rate of outcomes was generally low regardless of the range of BMI. Among patients with elevated GDF-15, the incidence rate was about 2-fold higher in those with low BMI than in those with normal or high BMI (Table 2). The detail cause of death was shown in Supplementary Table 1.

**Table 2:** Number and incidence rate of outcomes.

**Outcome**	**Parameters**	**Overall**	**Non-elevated GDF-15 with normal BMI**	**Non-elevated GDF-15 with high BMI**	**Non-elevated GDF-15 with low BMI**	**Elevated GDF-15 with normal BMI**	**Elevated GDF-15 with high BMI**	**Elevated GDF-15 with low BMI**
		***n* = 4,061**	**(Group A)**	**(Group B)**	**(Group C)**	**(Group D)**	**(Group E)**	**(Group F)**
			***n* = 885**	***n* = 1,035**	***n* = 834**	***n* = 428**	***n* = 459**	***n* = 420**
All-cause death	No. of events	174 (4.3%)	23 (2.6%)	21 (2.0%)	18 (2.2%)	27 (6.3%)	34 (7.4%)	51 (12.1%)
	Incidence rate, 1,000 person-year	6.7	3.9	3.1	3.2	10.4	11.8	22.1
Cancer death	No. of events	68 (1.7%)	11 (1.2%)	6 (0.6%)	8 (1.0%)	8 (1.9%)	12 (2.6%)	23 (5.5%)
	Incidence rate, 1,000 person-year	2.6	1.9	0.9	1.4	3.1	4.2	9.9
CV death	No. of events	56 (1.4%)	9 (1.0%)	11 (1.1%)	2 (0.2%)	10 (2.3%)	10 (2.2%)	14 (3.3%)
	Incidence rate, 1,000 person-year	2.2	1.5	1.6	0.4	3.8	3.5	6.1

*GDF-15, growth differentiation factor-15; BMI, body mass index; CV death, cardiovascular death*.

**Figure 1:**
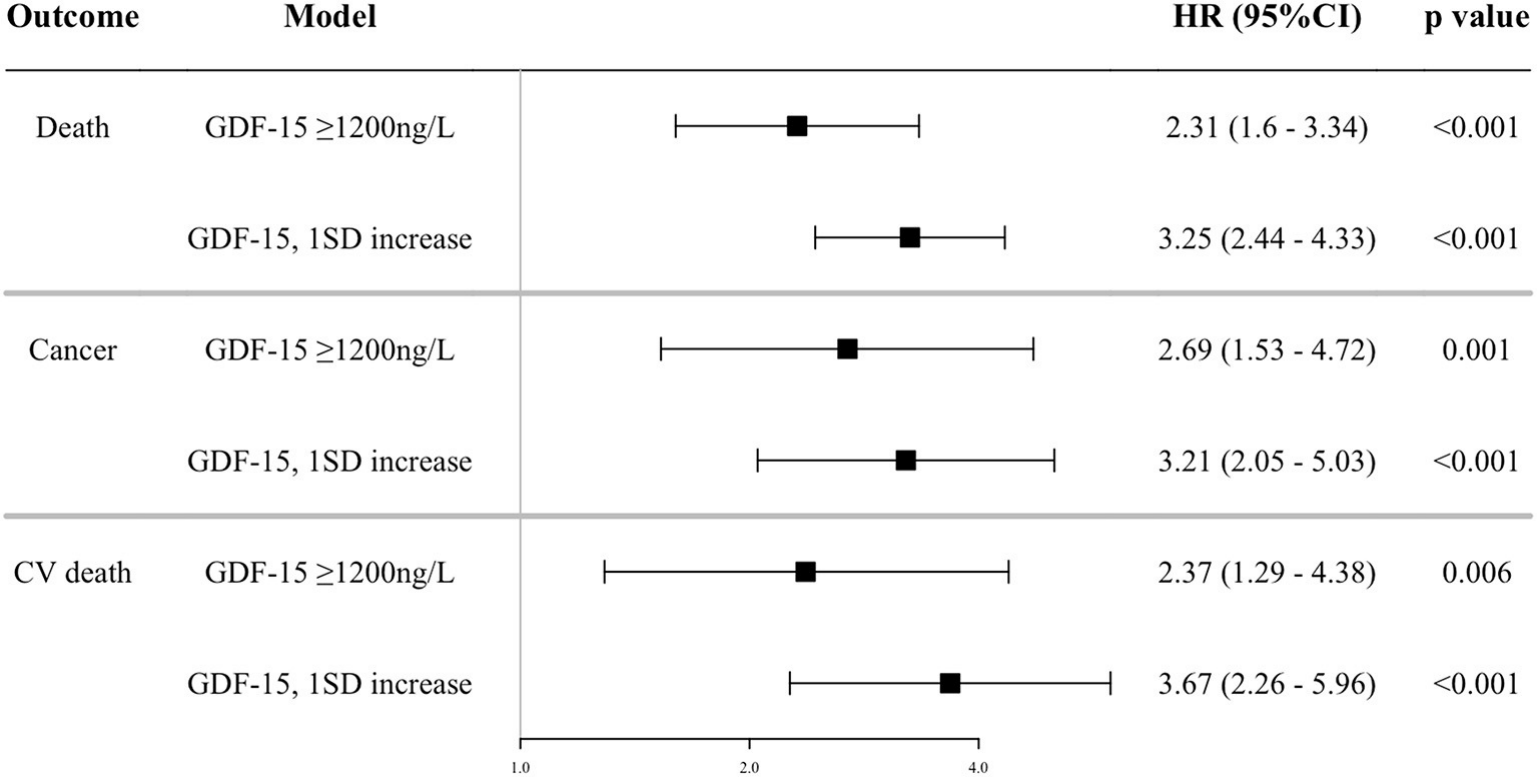
Association between GDF-15 and outcomes. Shown are the results of a Cox proportion hazard analysis by continuous and dichotomous models of GDF-15 and outcomes adjusted for the composite risk score of CV traditional risk factors (age, sex, current smoking, diabetes mellitus, previous CV event, statin use, anti-hypertensive drug use, total cholesterol, high-density lipoprotein cholesterol, office systolic blood pressure), estimated glomerular filtration rate, daily drinker, and high sensitive C reactive protein (log). GDF-15, growth differentiation factor-15; CV death, cardiovascular death; HR, hazard ratio; 95%CI, 95% confidence interval.

In multivariable Cox proportional hazards models adjusted for traditional risk factors, higher concentrations of GDF-15 modeled as a dichotomous (relative to <1,200 ng/L) and continuous variable (1SD increase) were associated with an increased risk for each outcome (Figure 1). In sensitive analyses, patients with ≥1,200 ng/L of GDF-15 were at a marginally increased risk of CV death compared to those with non-elevated GDF-15 (HR 1.80, 95%CI 0.95–3.44, *p* = 0.073, Supplementary Figure 1). Not discrimination (c-statistics) but reclassification (NRI and IDI) was significantly improved with the addition of log GDF-15 to the fully adjusted model for all-cause, cancer, and CV death (Table 3). In sensitive analyses adjusted for individual covariates, incorporating log GDF-15 improved discrimination (c-statistics) of the model for each outcome (Supplementary Table 2). These findings suggested that GDF-15 was not only associated with each outcome but was also a sufficiently strong predictor to exhibit an incremental benefit for the model including the traditional risk factors.

**Table 3:** Change in risk-predictive metrics by incorporating prognostic biomarkers to the base model.

	**c-statistics (95%CI)**	**Difference of c-statistics (95%CI)**	**Category-free NRI (95%CI)**	**IDI (95%CI)**
**All-cause death**				
Model 1	0.768 (0.729–0.807)			
Model 1 + log GDF-15	0.790 (0.755–0.826)^§^	0.022 (0.004–0.043)	0.251 (0.147–0.344)^‡^	0.033 (0.014–0.058)^‡^
**Cancer death**				
Model 1	0.753 (0.682–0.824)			
Model 1 + log GDF-15	0.781 (0.721–0.842)	0.028 (−0.005–0.068)	0.215 (0.062–0.371)^*^	0.019 (0.004–0.049)^†^
**CV death**				
Model 1	0.757 (0.689–0.826)			
Model 1 + log GDF-15	0.784 (0.720–0.847)	0.026 (−0.010–0.068)	0.361 (0.142–0.465)^†^	0.023 (0.005–0.061)^†^

*Model 1 was adjusted for the composite risk score of CV traditional risk factors (age, sex, current smoking, diabetes mellitus, previous CV event, statin use, anti-hypertensive drug use, total cholesterol, high-density lipoprotein cholesterol, office systolic blood pressure), estimated glomerular filtration rate, daily drinker, and high sensitive C reactive protein (log). The 95% confidence intervals (CIs) of each metric were estimated by using 1,000 bootstrap samples*.

* *p < 0.05,*

† *p < 0.01, and*

‡ *p < 0.001.*

§ *Significant improvement of c-statistics, i.e., the 95%CIs were not <0. GDF-15, growth differentiation factor-15; BMI, body mass index; CV death, cardiovascular death; NRI, net reclassification improvement; IDI, integrated discrimination improvement*.

### High Prognostic Value of GDF-15 in Patients With Low BMI

**Figure 2:**
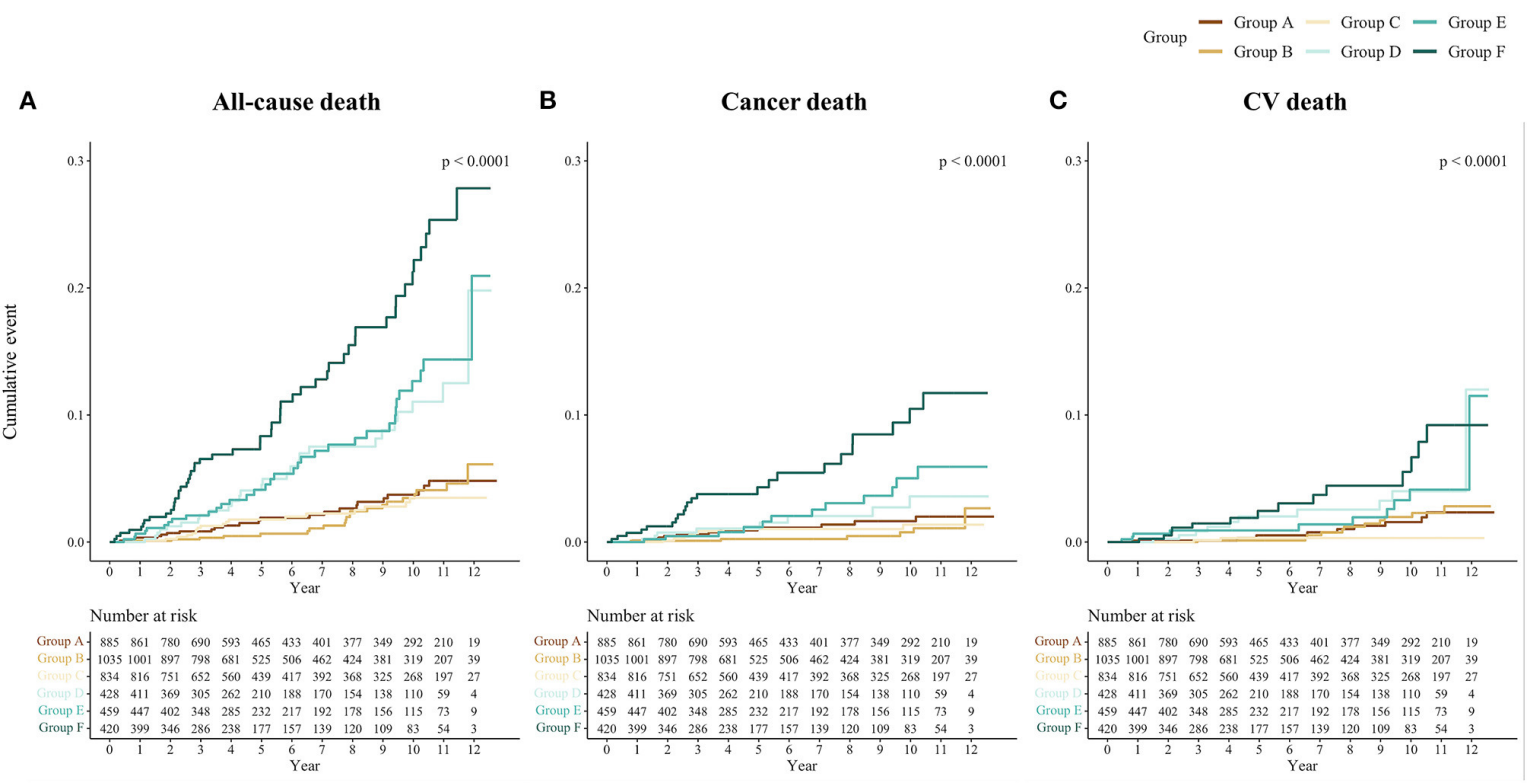
Cumulative incidence by outcomes and the GDF-15/BMI-based groups. Kaplan-Meier curves of the cumulative incidence rates of **(A)** all-cause, **(B)** cancer, and **(C)** CV death by the GDF-15/BMI-based study group are shown. The GDF-15/BMI-based study groups were as follows: Group A, non-elevated GDF-15 (<1,200 ng/L) with normal BMI (22.5–25.0 kg/m^2^); Group B, non-elevated GDF-15 with high BMI (≥25.0 kg/m^2^); Group C, non-elevated GDF-15 with low BMI (<22.5 kg/m^2^); Group D, elevated GDF-15 (≥1,200 ng/L) with normal BMI; Group E, elevated GDF-15 with high BMI; and Group F, elevated GDF-15 with low BMI. GDF-15, growth differentiation factor-15; BMI, body mass index; CV death, cardiovascular death.

**Figure 3:**
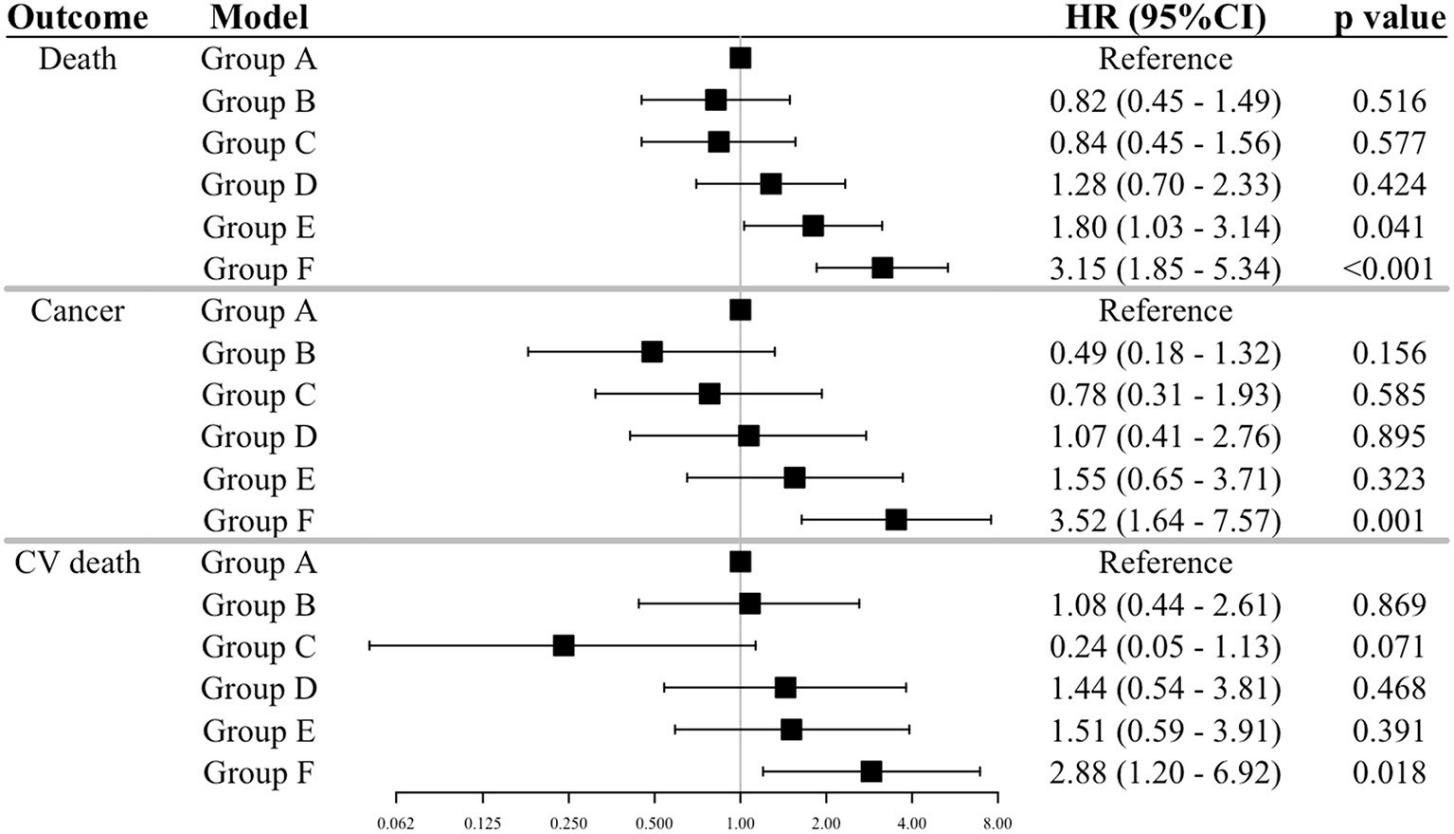
Relations of the GDF-15/BMI-based groups to outcomes. The results of a Cox proportion hazard analysis by the GDF-15/BMI-based group outcomes adjusted for traditional risk factors are shown. The GDF-15/BMI-based groups were as defined in Figure 2. Adjustments of the Cox model are as shown in Figure 1. GDF-15, growth differentiation factor-15; BMI, body mass index; CV death, cardiovascular death.

The cumulative Kaplan-Meier plots of each outcome by the GDF-15/BMI-based study groups are shown in Figure 2. In the groups with non-elevated GDF-15 (Groups A, B, and C), the incidence of each outcome was generally low, and patients with elevated GDF-15 and low BMI (Group F) were associated with increased event rates during follow-up. The event rates of patients with elevated GDF-15 and normal or high BMI (Groups D and C) showed an intermediate separation of event curves. Patients with elevated GDF-15 and low BMI (Group F) were at significantly increased risk for each outcome compared with the reference group (Group A) in multivariable Cox proportional hazards models adjusted for traditional risk factors (Figure 3).

**Figure 4:**
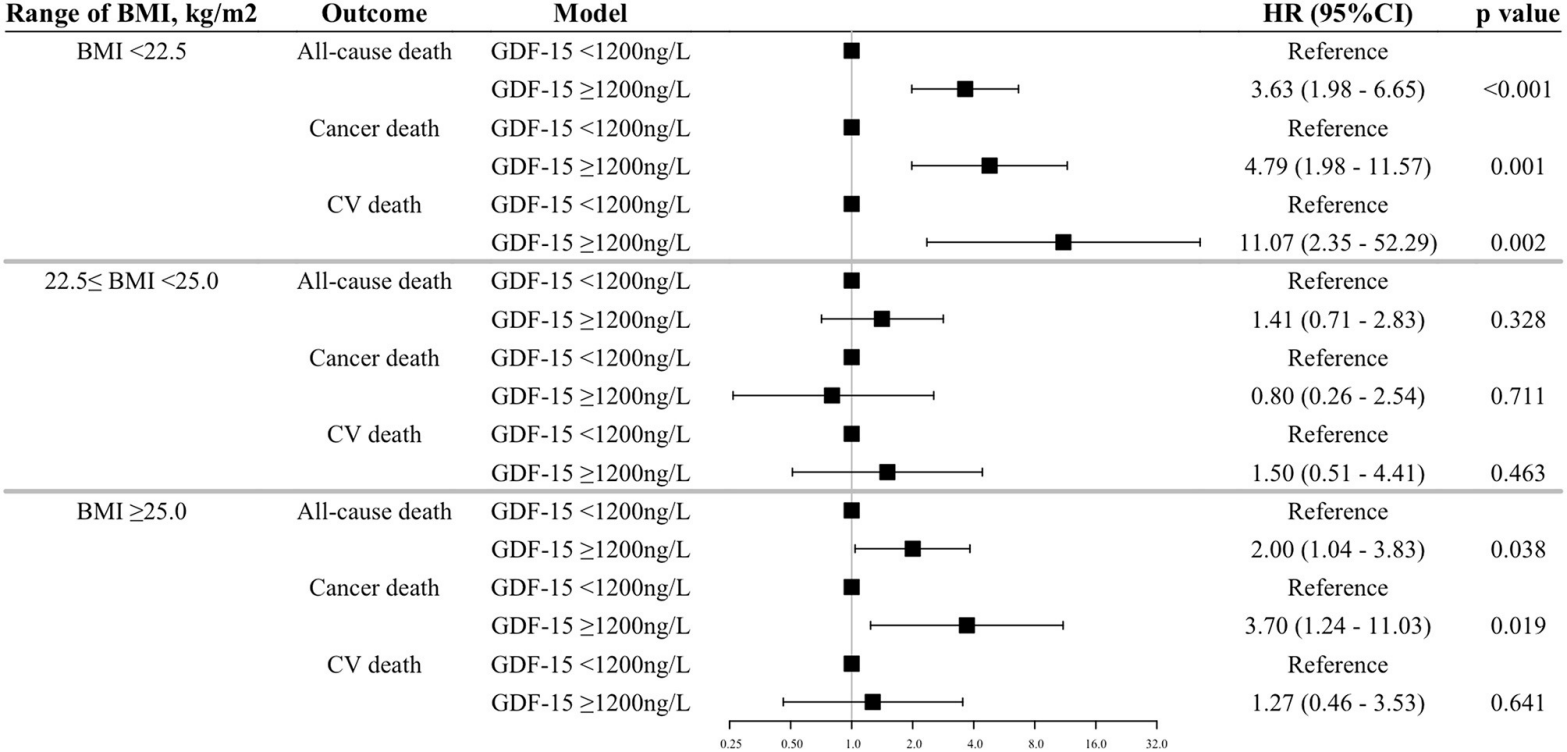
Association between elevated GDF-15 and outcomes in the BMI subgroups. Shown are the results of a Cox proportion hazard analysis by a dichotomous model of GDF-15 and outcomes adjusted for traditional risk factors. The BMI subgroups were stratified based on the cut-off points of 22.5 and 25.0 kg/m^2^ (i.e., <22.5 as low BMI, 22.5–25.0 as normal BMI, ≥25.0 as high BMI). Adjustments of the Cox model are as shown in Figure 1. GDF-15, growth differentiation factor-15; BMI, body mass index; CV death, cardiovascular death.

Next, we analyzed the relationship between GDF-15 and outcomes in each range of BMI (i.e., <22.5, 22.5–25.0, ≥25.0 kg/m^2^). In the BMI range of <22.5 kg/m^2^, patients with elevated GDF-15 were associated with increasing hazard for all-cause death and more than a 4-fold increased risk of cancer and CV death as compared with those with non-elevated GDF-15. The relationship between GDF-15 and outcomes was also supported in the groups of high BMI, with a consistently increasing risk of all-cause and cancer death (except CV death) with elevated GDF-15 levels (Figure 4). On the other hand, in the range of normal BMI, elevated GDF-15 levels were not associated with outcomes after adjustment for traditional risk factors (Figure 4). In the sensitive analyses, we got the similar results, however, the association of GDF-15 with the risk of all-cause and cancer death became no longer statistically significant in the groups of high BMI (Supplementary Figure 3).

We also analyzed the relations of GDF-15 level to outcome in other subgroups based on the BMI cut-off points (21 and 27 kg/m^2^) from a Japanese cohort study (29). The associations between elevated GDF-15 and the increased risks of outcomes were also limited in the range under 21 kg/m^2^ of BMI (all-cause death, HR 3.65, 95%CI 1.63–8.21, *p* = 0.002; cancer death HR 4.86, 95%CI 1.38–17.14, *p* = 0.014; and CV death, HR 18.35, 95%CI 2.24–150.1, *p* = 0.007, relative to the reference group with non-elevated GDF-15, respectively). These findings suggested that the high prognostic value of GDF-15 was limited in patients with low BMI.

## Discussion

The present results showed that elevated GDF-15 was associated with increased risks of all-cause, cancer, and CV death in outpatients with CV risk factors, and that incorporating GDF-15 to the predictive model for these outcomes improved its performance significantly. In the sub-analyses stratified the range of BMI, elevated GDF-15 levels were associated with increased risks of all-cause and cancer death in the groups of low and high BMI. The relationship between GDF-15 and the risk of CV death was stronger only in the group with BMI <22.5 kg/m^2^. However, these prognostic values of GDF-15 were not observed in the subgroups with a normal range of BMI (22.5–25.0 kg/m^2^). These findings suggests that GDF-15 is a strong predictor for all-cause, cancer, and CV death in patients with CV risk factors and especially in those with low BMI.

Our study showed that GDF-15 had a high prognostic value for cancer death in patients with CV risk factors. GDF-15 is a stress-induced cytokine whose expression is induced by transcription factors such as p53 and EGR-1 (30, 31). Although GDF-15 is weakly expressed in the liver, kidney, and prostate, its expression from other tissues increases in the presence of stress or a disease state (4, 5). In common cancers, including breast, colon, prostate, and pancreas cancers, GDF-15 is produced in tumor tissues (6, 7, 32–34). GDF-15 is cleaved from a propeptide by furin-like proteases before its secretion, but this intracellular cleavage from a propeptide does not process efficiently in tumor tissue (6). The 3-h half-life of GDF-15 is prolonged in the circulation, and the serum levels of GDF-15 increase markedly in advanced cancer (7). In this study, patients with cancer were excluded at baseline, so the outcome of cancer death implied that the patients developed malignancy and died of advanced cancer. Given that the mean follow-up period was 6.6 years, patients who died from malignancy might have had undiagnosed cancer at baseline.

This study further found that high GDF-15 levels in patients with low BMI conferred an increased risk for all-cause death, cancer death, and CV death. In a cohort of patients with advanced cancer, serum GDF-15 levels were strongly correlated to weight loss (18), and patients with chronic renal and cardiac failure were also associated with anorexia-cachexia (16, 17). Based on recent developments in GDF-15-related research, the pathological mechanism underlying this association was considered to involve activation of the GFRAL/RET complex and the resulting effect on the appestat (12–15). GDF-15 molecules bind to GFRAL receptors, and this complex combines two RET molecules. Then, thte GDF-GFRAL-RET complex signals downstream effectors including Akt, ERK1/2, and PLCγ, and activates c-Fos in the area postrema (AP) of the hindbrain and solitary nucleus (35, 36). The activation of AP also activates other brain centers such as the hypothalamus, parabrachial nucleus, and amygdala, suggesting effects on complex feeding and emotional behaviors (13, 16). In addition, this physiological mechanism is independent of other pathways which affect feeding, such as melanocortin-4 and leptin receptors (13). These evidences suggest that GDF15-GFRAL-RET is a central regulator of feeding and body weight and a new therapeutic target of both anorexia-cachexia and obesity (14, 36, 37). Body weight loss resulting from elevated GDF-15 might cause poor prognosis in patients with various types of cancer and CVD. In sub-analyses of groups with low BMI, patients with non-elevated GDF-15 levels (group C) were considered to have relatively low risks for anorexia-cachexia and were not associated with poor prognosis. On the other hand, patients with elevated GDF-15 levels (group F), who might be at risk of anorexia-cachexia, had more than 4-fold elevation of risk for cancer and CV death compared with those with non-elevated GDF-15 (Group C). These facts suggest that the serum GDF-15 levels of patients with low BMI would be useful as a marker to identify weight loss sufficient to cause poor prognosis in various diseases.

Several large pooled analyses indicated a U-shaped relationship between BMI and increased risks of all-cause, cancer, and CV death (23, 24, 29, 38, 39), and also reported strong associations between lower BMI and death from respiratory disease and between higher BMI and cancer and CV death (24, 38). In this study, the BMI range of <22.5 kg/m^2^ was associated with an increased risk of all-cause and cancer death compared with that of 22.5–25.0 kg/m^2^ in a Cox hazard model, however, the BMI range of ≥25.0 kg/m^2^ was not associated with an increased risk of all-cause, cancer, and CV death (data not shown). This finding may have been related to our study design, which included an older population and a shorter follow-up period compared to previous large cohorts. In particular, patients in this study were undergoing treatment for primary prevention of CVD, which naturally could have decreased the incidence of CV death.

There were some limitations of this study. First, we measured blood GDF-15 levels only one time at the baseline, and thus we could not assess the interaction and fluctuation of GDF-15 levels over time during the progression of each adverse event. Second, the patients in this study were all Japanese, and thus our findings may not be generalizable to other race/ethnic groups. Third, the study outcomes were cancer and CVD mortality, so we could not assess the prognostic value of GDF-15 for the development of cancer and CVD in this study. Additionally, many of the records of cancer death did not mention the primary lesion (Supplementary Table 1), we could not assess which type of cancers developed. Fourth, variables which affected BMI were limited to alcohol usage. It was preferable to add the adjustments related body weight, such as poor nutrition and physical inactivity. This issue might affect the results potentially.

In conclusion, GDF-15 might be a strong predictor for all-cause, cancer, and CV death in patients with CV risk factors. Especially in patients with low BMI, elevated GDF-15 levels were related to higher risks for these outcomes. Because elevated GDF-15 levels could cause body weight loss through effects on the appestat, serum GDF-15 levels might be a useful marker to identify the potential anorexia-cachexia in patients with low BMI.

## Data Availability Statement

The datasets generated for this article are not readily available because our group plans to submit articles continuously by using the datasets. Requests to access the datasets should be directed to kkario@jichi.ac.jp.

## Ethics Statement

The studies involving human participants were reviewed and approved by Institutional Review Board of Jichi Medical School. The patients/participants provided their written informed consent to participate in this study.

## Author Contributions

KK, SH, and MS contributed to conception and design of the study. SH organized the database. KN performed the statistical analysis and wrote the first draft of the manuscript. All authors contributed to manuscript revision, read, and approved the submitted version.

## Conflict of Interest

KK has received research grants and honoraria from Roche Diagnostics, Omron Healthcare and A& D Co. The remaining authors declare that the research was conducted in the absence of any commercial or financial relationships that could be construed as a potential conflict of interest.

## Supplementary Material

Supplementary Figure 1 Association between GDF-15 and outcomes by Cox proportion hazard analyses adjusted for the individual covariates. Shown are the results of a Cox proportion hazard analysis by continuous and dichotomous models of GDF-15 and outcomes adjusted for age, sex, current smoking, diabetes mellitus, previous CV event, statin use, anti-hypertensive drug use, total cholesterol, high-density lipoprotein cholesterol, office systolic blood pressure, estimated glomerular filtration rate, daily drinker, and high sensitive C reactive protein (log). GDF-15, growth differentiation factor-15; CV death, cardiovascular death; HR, hazard ratio; 95%CI, 95% confidence interval.

Supplementary Figure 2 Relations of the GDF-15/BMI-based groups to outcomes by Cox proportion hazard analyses adjusted for the individual covariates. The results of a Cox proportion hazard analysis by the GDF-15/BMI-based group outcomes adjusted for traditional risk factors are shown. The GDF-15/BMI-based study groups were as follows: Group A, non-elevated GDF-15 (<1,200 ng/L) with normal BMI (22.5–25.0 kg/m^2^); Group B, non-elevated GDF-15 with high BMI (≥25.0 kg/m^2^); Group C, non-elevated GDF-15 with low BMI (<22.5 kg/m^2^); Group D, elevated GDF-15 (≥1,200 ng/L) with normal BMI; Group E, elevated GDF-15 with high BMI; and Group F, elevated GDF-15 with low BMI. Adjustments of the Cox model are as shown in Supplementary Figure 1. GDF-15, growth differentiation factor-15; BMI, body mass index; CV death, cardiovascular death.

Supplementary Figure 3 Association between elevated GDF-15 and outcomes in the BMI subgroups by Cox proportion hazard analyses adjusted for the individual covariates. Shown are the results of a Cox proportion hazard analysis by a dichotomous model of GDF-15 and outcomes adjusted for traditional risk factors. The BMI subgroups were stratified based on the cut-off points of 22.5 and 25.0 kg/m^2^ (i.e., <22.5 as low BMI, 22.5–25.0 as normal BMI, ≥25.0 as high BMI). Adjustments of the Cox model are as shown in Supplementary Figure 1. GDF-15, growth differentiation factor-15; BMI, body mass index; CV death, cardiovascular death.

Supplementary Table 1 Cause of death.

Supplementary Table 2 Change in risk-predictive metrics by incorporating prognostic biomarkers to the base modeladjusted for the individual covariates.
