# Supplementary material for: Growth Differentiation Factor 15 Predicts Cancer Death in Patients With Cardiovascular Risk Factors: The J-HOP Study
Source: Front Cardiovasc Med. 2021 Jun 4;8:660317. doi: 10.3389/fcvm.2021.660317 (PMC8211884; doi:10.3389/fcvm.2021.660317)

Supplementary figure 1. Association between GDF-15 and outcomes

Outcome	Model	HR (95%CI)	p value
Death	GDF-15 ≥1200ng/L	1.88 (1.30 - 2.71)	0.001
	GDF-15, 1SD increase	2.89 (2.13 - 3.92)	<0.001
Cancer	GDF-15 ≥1200ng/L	2.06 (1.15 - 3.67)	0.014
	GDF-15, 1SD increase	2.78 (1.73 - 4.45)	<0.001
CV death	GDF-15 ≥1200ng/L	1.80 (0.95 - 3.44)	0.073
	GDF-15, 1SD increase	3.23 (1.87 - 5.57)	<0.001

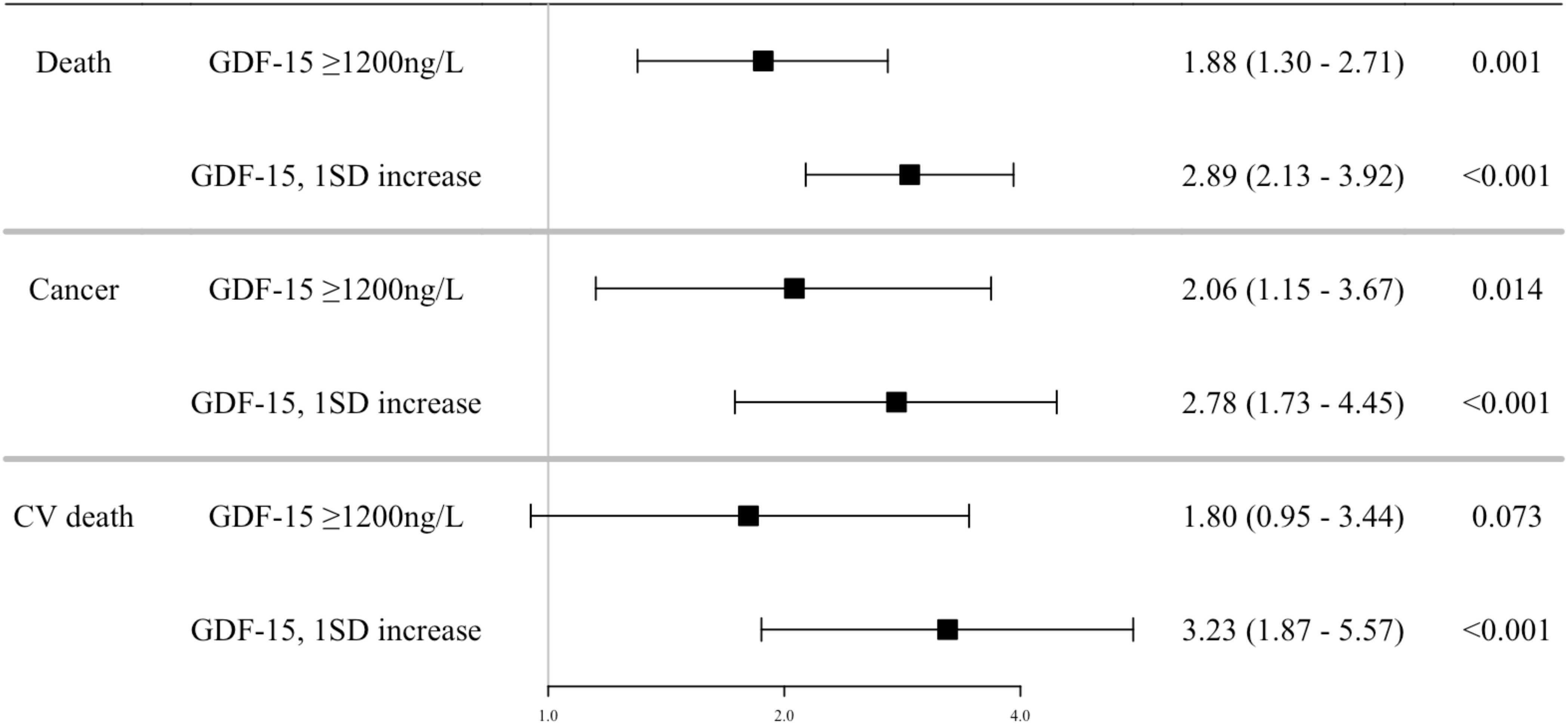

Supplementary figure 2. Relations of the GDF-15/BMI-based groups to outcomes

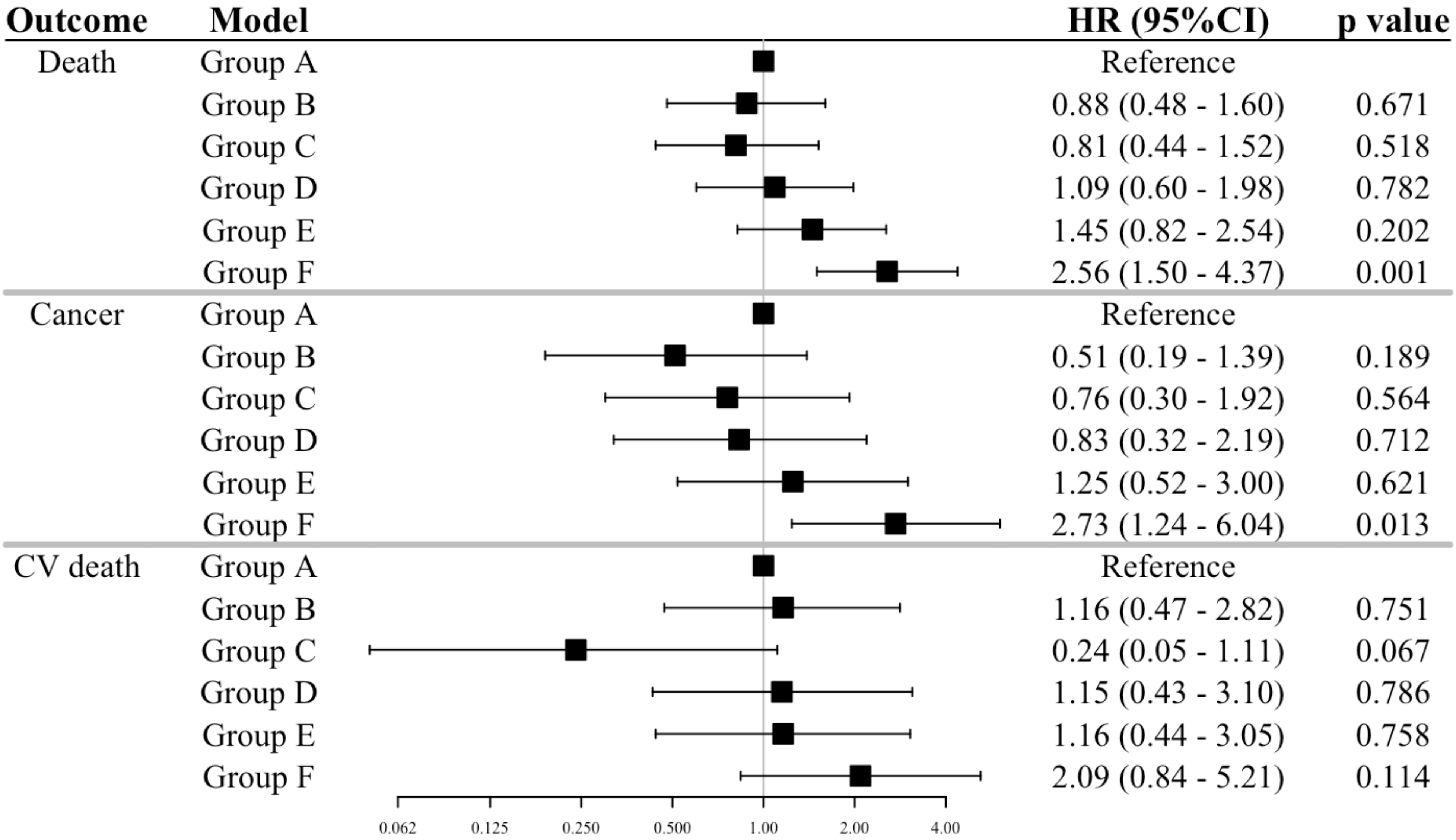

Supplementary figure 3. Association between elevated GDF-15 and outcomes in the BMI subgroups

Range of BMI, kg/m2	Outcome	Model	HR (95%CI)	p value
BMI <22.5	All-cause death	GDF-15 <1200ng/L	Reference	
		GDF-15 ≥1200ng/L	2.75 (1.50 - 5.05)	0.001
	Cancer death	GDF-15 <1200ng/L	Reference	
		GDF-15 ≥1200ng/L	3.99 (1.57 - 10.14)	0.004
	CV death	GDF-15 <1200ng/L	Reference	
		GDF-15 ≥1200ng/L	4.80 (0.99 - 23.16)	0.051
22.5≤ BMI <25.0	All-cause death	GDF-15 <1200ng/L	Reference	
		GDF-15 ≥1200ng/L	1.19 (0.59 - 2.40)	0.635
	Cancer death	GDF-15 <1200ng/L	Reference	
		GDF-15 ≥1200ng/L	0.67 (0.22 - 2.06)	0.486
	CV death	GDF-15 <1200ng/L	Reference	
		GDF-15 ≥1200ng/L	1.14 (0.35 - 3.75)	0.825
BMI ≥25.0	All-cause death	GDF-15 <1200ng/L	Reference	
		GDF-15 ≥1200ng/L	1.64 (0.85 - 3.18)	0.142
	Cancer death	GDF-15 <1200ng/L	Reference	
		GDF-15 ≥1200ng/L	2.44 (0.76 - 7.89)	0.136
	CV death	GDF-15 <1200ng/L	Reference	
		GDF-15 ≥1200ng/L	1.01 (0.34 - 3.04)	0.981

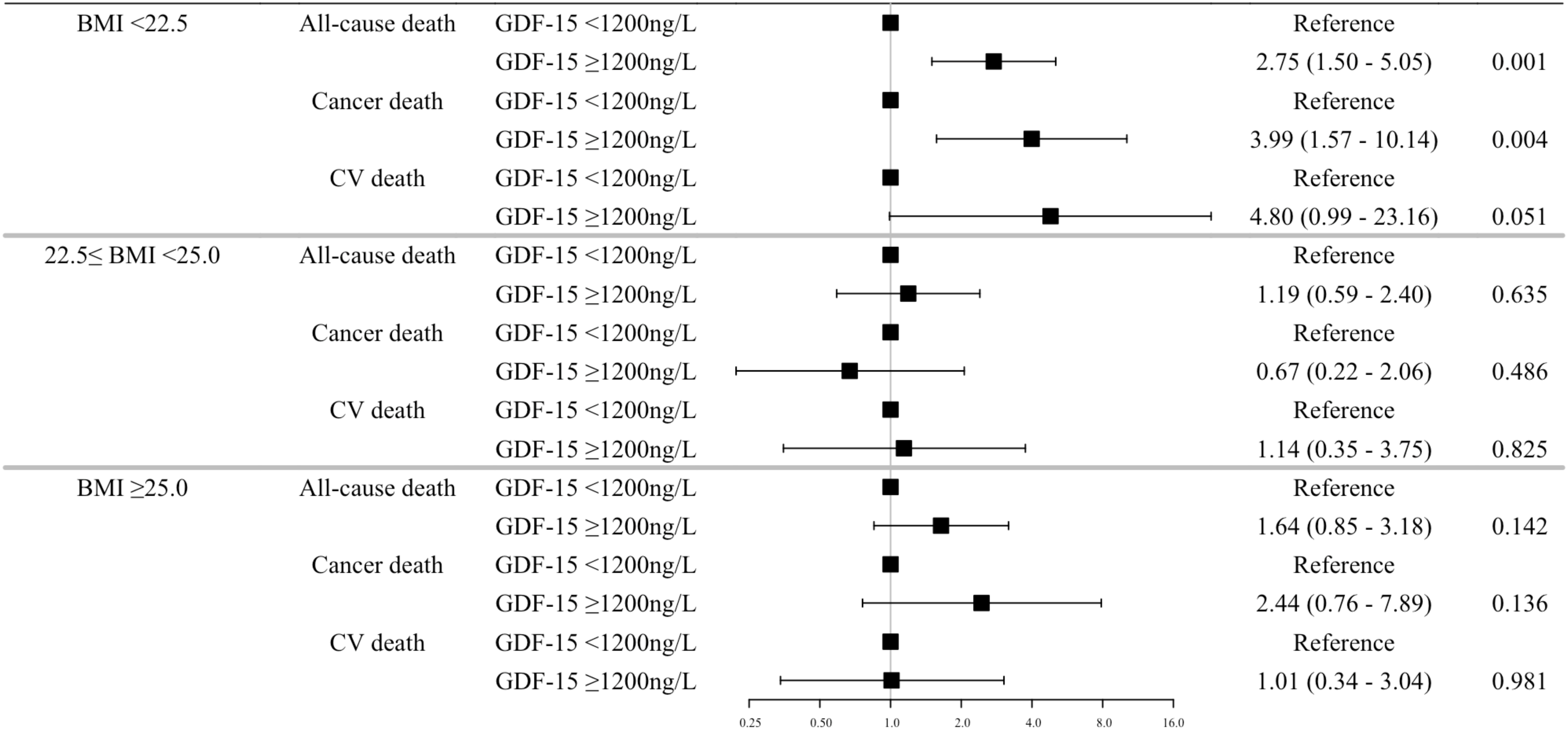

Supplement: Supplementary Figure 1 — Association between GDF-15 and outcomes by Cox proportion hazard analyses adjusted for the individual covariates. Shown are the results of a Cox proportion hazard analysis by continuous and dichotomous models of GDF-15 and outcomes adjusted for age, sex, current smoking, diabetes mellitus, previous CV event, statin use, anti-hypertensive drug use, total cholesterol, high-density lipoprotein cholesterol, office systolic blood pressure, estimated glomerular filtration rate, daily drinker, and high sensitive C reactive protein (log). GDF-15, growth differentiation factor-15; CV death, cardiovascular death; HR, hazard ratio; 95%CI, 95% confidence interval. [file Data_Sheet_2.pdf]
