# Supplementary material for: Growth Differentiation Factor 15 Predicts Cancer Death in Patients With Cardiovascular Risk Factors: The J-HOP Study
Source: Front Cardiovasc Med. 2021 Jun 4;8:660317. doi: 10.3389/fcvm.2021.660317 (PMC8211884; doi:10.3389/fcvm.2021.660317)
Supplement: Supplementary Table 1 — Cause of death. [file Data_Sheet_1.pdf]

Supplementary table 1. Cause of death.

**Cause of death**	**Cause of death**	**Number (share, %)**
**Cancer death**	Lung cancer	9 (5.17%)
	Gastric cancer	4 (2.3%)
	Esophageal cancer	4 (2.3%)
	Liver cancer	4 (2.3%)
	Other	8 (4.6%)
	Not Recorded	39 (22.41%)
**CV death**	Myocardial infarction	9 (5.17%)
	Heart failure	14 (8.05%)
	Stroke	16 (9.2%)
	Sudden death	17 (9.77%)
**Non CV death**	Pneumonia	11 (6.32%)
	Hepatic insufficiency	4 (2.3%)
	Sepsis	4 (2.3%)
	Suicide	5 (2.87%)
	Other	16 (9.2%)
	Unknown	10 (5.75%)

Other of Cancer death includes pancreatic cancer, breast cancer, leukemia, and malignant lymphoma. Sudden death includes ventricular fetal arrhythmias and aortic dissection. Other of Non CV death includes gastrointestinal bleeding, adrenal insufficiency, and accidents. Abbreviations: CV death, cardiovascular death.

Supplementary table 2. Change in risk-predictive metrics by incorporating prognostic biomarkers to the base model adjusted for the individual covariates.

	c-statistics (95%CI)	Difference of c-statistics (95%CI)	Category-free NRI (95%CI)	IDI (95%CI)
**All-cause death**				
Model 1	0.780 (0.744 – 0.817)			
Model 1 + log GDF-15	0.795 (0.759 – 0.830)§	0.014 (0.004 – 0.027)	0.182 (0.077 – 0.278)‡	0.028 (0.009 – 0.054)‡
**Cancer death**				
Model 1	0.775 (0.715 – 0.834)			
Model 1 + log GDF-15	0.793 (0.735 – 0.850)§	0.018 (0.001 – 0.039)	0.133 (-0.010 – 0.286)	0.016 (0.000 – 0.049)*
**CV death**				
Model 1	0.781 (0.719 – 0.843)			

Model 1 + log GDF-15	0.797 (0.737 – 0.858)§	0.016 (0.000 – 0.039)	0.296 (0.083 – 0.430)†	0.017 (0.002 – 0.064)*

Model 1 was adjusted for age, sex, current smoking, diabetes mellitus, previous CV event, statin use, anti-hypertensive drug use, total cholesterol, high-density lipoprotein cholesterol, office systolic blood pressure, estimated glomerular filtration rate, daily drinker, and high sensitive C reactive protein (log). The 95% confidence intervals (CIs) of each metric were estimated by using 1,000 bootstrap samples. *p<0.05, †p<0.01, and ‡p<0.001. §Significant improvement of c-statistics, i.e., the 95%CIs were not <0. Abbreviations: GDF-15, growth differentiation factor-15; BMI, body mass index; CV death, cardiovascular death; NRI, net reclassification improvement; IDI, integrated discrimination improvement.
